# Combustion of Pneumoperitoneum: A Rare Complication Secondary to Spontaneous Combustion of Free Gas in the Peritoneal Cavity

**DOI:** 10.7759/cureus.28962

**Published:** 2022-09-08

**Authors:** Veezhmhan Seynulabdeen

**Affiliations:** 1 Anaesthesiology, Base Hospital Dambadeniya, Colombo, LKA

**Keywords:** theatre fire, diathermy, ignition, laparotomy, pneumoperitoneum

## Abstract

We report a rare incident of explosive combustion of free gas in the peritoneal cavity when an electrosurgery electrode was used to enter the peritoneal cavity via a midline incision. Pneumoperitoneum was due to spontaneous jejunal perforation. No injury was caused to the patient or medical personnel. Theatre staff should be aware of the possible risk of combustion of bowel gas during similar scenarios. It is prudent to use sharp dissection when entering the peritoneal cavity, and cautery can be used once the free air gas escapes from the peritoneal cavity.

## Introduction

To produce fire, the presence of an oxidiser, fuel and an ignition source is required. Oxygen and nitrous oxide are the two oxidisers present in the operating theatre, while surgical spirit, drapes and gastrointestinal gas can serve as fuel [1]. Diathermy and laser are the commonly discussed ignition sources [2]. Operating theatre fire incidents are uncommon [1,2]. Head and neck surgeries and facial surgeries are the most commonly involved surgeries in theatre fire accidents, and the involvement of abdominal surgeries in such accidents is rare [3].

## Case presentation

**Figure 1:**
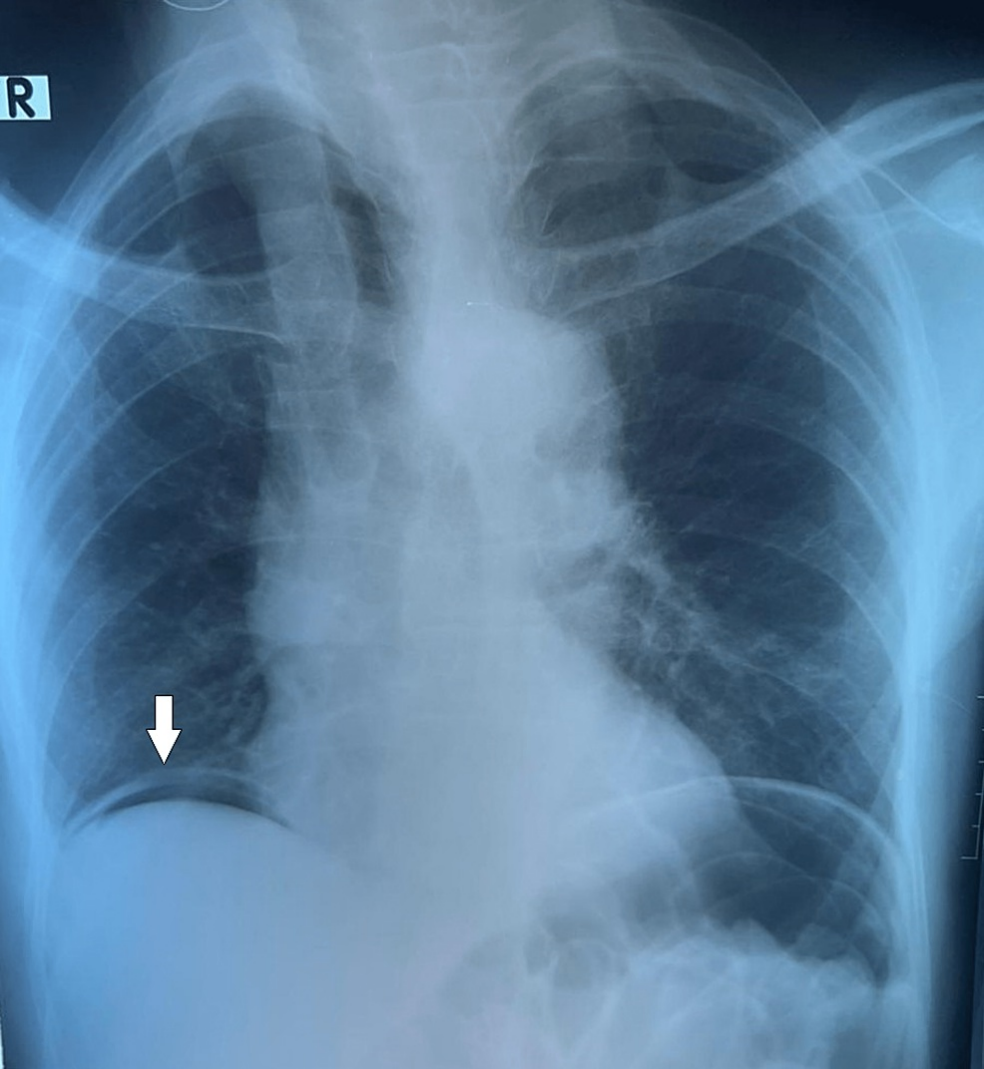
Erect chest radiograph demonstrating sub-diaphragmatic free air (white arrow).

A 79-year-old male presented to the emergency treatment unit with a one-day history of severe epigastric pain. There was no history of altered bowel habits, vomiting, gastro-oesophageal reflux disease, or previous abdominal surgeries. He was on aspirin, clopidogrel, losartan, nifedipine and atorvastatin for hypertension, ischaemic heart disease and dyslipidaemia. On examination, his abdomen was rigid and tender for soft palpation and he was haemodynamically stable with a Glasgow Coma Scale score of 15/15. An urgent erect chest radiograph revealed pneumoperitoneum (Figure 1) suggestive of a hollow viscus perforation. The patient was taken to an urgent explorative laparotomy while being resuscitated.

The patient was pre-oxygenated with 100% oxygen through the face mask until the end-tidal oxygen was 90% before undergoing routine rapid sequence induction. Anaesthesia was maintained with 40% oxygen and 1% isoflurane. The abdomen was prepared with 10% povidone-iodine and draped with square surgical drapes. Midline laparotomy skin incision was made using a scalpel and continued with monopolar diathermy until the peritoneal cavity is opened into. Once the peritoneal cavity was entered using the diathermy (50/50), a loud ‘pop’ was heard with a small momentary fireball. The laparotomy was paused for a few minutes, there were no evident injuries to the patient or the surgeon and the laparotomy progressed. A localised 0.5 cm perforation was found at the distal part of the jejunum, with contamination of the peritoneal cavity with enteric contents. The defect was repaired, the peritoneal cavity was thoroughly washed and routine closure was done after placing a drain. The patient recovered from anaesthesia without any complications and was transferred to the high-dependency unit for post-operative monitoring.

## Discussion

Inflammable gases are produced in the human gastrointestinal tract. Hydrogen and methane are the two common gases produced in the bowel. The degree of production of these gases changes with diet, digestion and metabolism [4]. The concentration of flammable gases is reduced with fasting for 12-24 hours, a low-residue diet and adequate bowel preparation [5].

A minimum of 5% of oxygen concentration is required for the combustion of bowel gas. Oxygen concentration in the gastrointestinal tract varies from 10% in the stomach to 5% in the distal colon. However, this may change with pre-oxygenation and delivery of supplementary oxygen during anaesthesia [1].

## Conclusions

In this case, the fuel leading to ignition was the bowel gas accumulated in the peritoneum and the ignition source was the handheld diathermy. Similar incidents of ignition of the bowel were reported that resulted in serious injuries to the patient and the surgeon. Therefore, we recommend the use of a scalpel to enter the peritoneal cavity in similar scenarios, and the use of electrosurgery is better avoided until the free gas accumulated in the peritoneal cavity is allowed to escape.

## References

[1] Dhebri AR, Afify SE (2002). Free gas in the peritoneal cavity: the final hazard of diathermy. Postgrad Med J.

[2] McSweeney WT, Kirkby B (2019). Combustion of pneumoperitoneum: a rare danger in the operating room. J Surg Case Rep.

[3] Shinagawa N, Mizuno H, Shibata Y, Yura J, Katsumi K, Ito M, Takeuchi T (1985). Gas explosion during diathermy colotomy. Br J Surg.

[4] Kirk E (1949). The quantity and composition of human colonic flatus. Gastroenterology.

[5] Avgerinos A, Kalantzis N, Rekoumis G, Pallikaris G, Arapakis G, Kanaghinis T (1984). Bowel preparation and the risk of explosion during colonoscopic polypectomy. Gut.

